# Missouri Valley Veterinary Association

**Published:** 1900-02

**Authors:** 


### MISSOURI VALLEY VETERINARY ASSOCIATION.

THE twenty-second regular meeting of the Missouri Valley Veterinary Association was held in the parlors of the Hotel Donovan, South St. Joseph, Mo., on the evening of December 11, 1899, at 7.30 P.M., with the following officers and members present: Dr. Forbes (President), Stewart, Moore, Washburn, Sloan, Netherton, Wright, Patterson, and Ross Cooper, with Drs. Blackwell, Wilson, Steele, Goode, of St. Joseph, and A. T. Peters, from Lincoln, Nebraska, as visitors.

A varied and very interesting surgical clinic was held on the afternoon of the same day, from 2 to 5 o'clock, in the hospital of Dr. E. J. Netherton, St. Joseph, Mo., under the direction of Drs. Netherton and Patterson.

Routine business having been disposed of, the President called upon Dr. Netherton, who read a paper on "Municipal Milk and Meat-inspection." (See p. 71.)

In the absence of Dr. Thackaberry, the discussion which followed the reading of the paper was led by Dr. S. Stewart, of Kansas City, as follows:

Dr. Stewart: I had not expected to enter very much into the discussion of this subject, and have not noted it as closely as I should have done. I wish to say, however, that I was quite interested in the paper, and think such papers should be placed before the public as frequently as possible, so that it may be fully and correctly informed, and be guided accordingly. Most of our veterinary papers that are prepared for veterinary societies are not very well adapted for presentation in the public press. They are much inclined to be too technical, too scientific. This paper, in my judgment, would serve a most excellent purpose if published in the daily press of this city, and I do not doubt but that it would receive considerable publicity by quotation. The statements contained therein are unquestionably in line with papers that have been presented heretofore, and I trust that the Society will see to it that the paper receives that publication which will put it into the hands of those who need to be informed upon such topics.

There is a theory extant that the veterinarian should not so frame his thoughts that they serve as alarmist articles; that he should be cautious; that it is unfair and unjust to the public to spread abroad alarmist articles; that they disquiet the comfort of the home; that mothers and fathers become anxious and do not appreciate the substance upon which they must live. But some alarming statements must be placed before them or they will not awaken to their danger. I know of no way of awakening their latent understanding without it is done somewhat rudely; so many things press for attention that it is only those things that arouse curiosity or that appeal to their fear for their personal safety, personal vanity, or some other powerful sentiment, which are likely to awaken any interest whatever, and such articles as these will serve a good purpose, even though there be some here and there who may be seriously disquieted by the discovery that such things are possible. The public press teems with articles upon impurities in other things beside meat and milk, and the public is becoming awakened to the necessity for the inspection of all kinds of food.

I note that the paper fixes the salary at $1600. Now that is a fair salary, and certainly would attract capable men to take up the work. It is not usually the case, however, that municipal bodies fix so large a salary as that to begin with, but even though the salary provided by any ordinance that might be passed should seemingly be meagre, I believe it would justify a local veterinarian who has other means of livelihood in accepting such an office, and if by his ability he can demonstrate his usefulness there is little doubt but that within a reasonably short time the municipality would raise the salary to an adequate sum. Some men say if they cannot get a substantial salary they will not accept a sanitary office of that kind, thinking it is demeaning to their special qualifications, but experience should teach such that it is not customary in any city for a newly-created office along sanitary lines to be very well paid, especially in States where it is just being inaugurated. They should be content with a meagre salary, and should also be content, if appointed, with accomplishing apparently small results, gradually extending their influence and the value of their office. The public is likely to be offended with sudden onsets of officialism in any direction, and soon rebel and dispose of it entirely. There are, therefore, two sides to the question, and the conservative one, it seems to me, should prevail. I hope the paper will receive the publication to which I have alluded.

Dr. Wilson: I would like to ask the author of this paper, which he thinks is the more likely to produce the typhoid fever, diphtheria, etc., to which he referred—tuberculosis or the uncleanliness of which he spoke?

Dr. Netherton: Well, I think scarlet fever and diphtheria are more frequently contracted as a result of the impurities in the water and the adulteration, than the tubercular trouble.

Dr. Peters: I was interested in Dr. Netherton's paper, and I hold the same views that Dr. Stewart does. At the Iowa-Nebraska meeting Dr. Ramacciotti gave a very excellent talk on this subject. He gave the history of milk-inspection in Omaha, and throughout his talk you could see how he accustomed the municipality to the inspection. He did not achieve his object by the method of the alarmist; but with sound, convincing argument he accomplished a great many things. He admitted that the system was by no means perfect, but stated that he was in hopes of having passed a new ordinance which he had framed and which he believed would improve the service. Now, I am a firm believer in that way of doing things. The veterinarians of Lincoln have long felt that they should do something. They looked to the experiment station to take the initiatory step. The men engaged in the practice there had frequent consultations with the experiment station authorities, and we also had consultation with the city authorities, and we found that the time was not ripe, but the small corps of veterinarians there kept up their little fight on the quiet, and lately we have received invitations to talk to the City Improvement Society, which is composed of ladies. Before that body I had the pleasure of talking in a popular way regarding the cleanliness of the city, proper sanitary precautions, and incidentally touched upon the question of the inspection of milk. I told them of the number of cases of diseased cows the veterinarians had found, and how powerless they were, and it seems that this organization is going to do far more than we ever dreamed of. We were always accused by the politicians of looking for a job, especially during the hard times, that horses were cheap, and we had to look for other fields of labor. I just throw out this suggestion that if you have such a society here, try and secure an invitation to give them a popular talk, and you will not be sorry.

Apropos of Dr. Netherton's reply to Dr. Wilson, I wish to say that I do not believe the typhoid, diphtheria, or tubercular germs are conveyed to the milk so much by adulteration as by the use of improperly cleansed vessels and the existence of unsanitary conditions around the dairy.

Dr. Netherton: I desire to say further that I have been treating cattle in several of the dairies around this city, and in one case which I recall I was called to see a cow which had calved, I think, two weeks before. She still had retention of the placenta, and they were saving the milk of that cow. There was such an odor from the animal that it was difficult to stay in the stall where she was. They had a well within ten feet of the stable. They have two hundred cows in the stable, and it was at this well that they washed their milk vessels. I saw them do that, and in all probability the water which was used to adulterate the milk was obtained from that same well. Last year was a very hard year on the dairymen in this city as well as others. The cows were not calving, the milk supply was short, and the fact of adulteration was not denied, but if they used that water to adulterate their milk certainly everything was favorable to the propagation of the germs of disease and their dissemination among the consumers of milk.

Dr. Moore: I do not know that I can add anything to what has been said in regard to this paper. I certainly appreciate it very much, and agree with the doctor fully in his statements. I think that ofttimes milk becomes contaminated, even though fairly good sanitary conditions prevail in the dairy, and care is bestowed upon the cans. The cooling-boxes are often sufficiently contaminated, and scarlet fever or typhoid fever may exist in the family, and the milk may become contaminated in that way. I do not think we could very well say anything to unnecessarily alarm the public. Of course, it might cause some people to say we are alarmists, but to me the matter is so important and covers so much territory, is so essential to the welfare of the people, that it would be hard to say anything that would alarm the people beyond what they should be alarmed. But whether it would not be policy to go a little slow might be a question.

So far as the necessity for publishing or getting before the people this class of scientific literature is concerned, I think that cannot be too greatly emphasized. I have felt for a long time that our efforts as veterinarians were confined too much to preparing papers for the veterinarian, for the scientific, educated men, and not enough for the masses, and it seems to me that it would be a good thing if at every meeting we had at least one paper devoted to matters of vital interest to the people, prepared in such a way that it would be acceptable to the press, especially the local press in the sections of the country where the meetings are held. If we could reach the people through that source it would benefit both the people generally and the veterinary profession.

Dr. Sloan: I had hoped that the writer of this paper would give us a practical solution of this question of milk inspection. I have seen a little of that tried in different places, and it is just as well that we note the mistakes of others in order not to fall into the same ruts ourselves. For instance, in San Francisco they have a milk inspector. He went about the city collecting samples of milk from the dairymen, and from his report the city undertook to enforce the testing of the milk from the different dairies. As a consequence the city has a number of law suits on its hands, the dairymen having combined to oppose the testing ordinance, and at the last accounts I had the matter was still in the courts. In Chicago the veterinary society proposed that the city take up the matter and enforce the testing of all the dairy cows in the city every three months. The mistake they made there was in giving the public the idea that it was necessary to test the cows every three months. If they had fixed the time at six months or a year they might possibly have accomplished their object. The frequency of the inspection fostered the opposition of the people to the scheme, however, and it was not popular for that reason. However, the matter was well stirred up, and in that way some good no doubt was accomplished, as it is necessary, it seems to me, to agitate these matters in order to educate the people to a sense of what is due them.

I think it would be a good thing to bring the matter before the physicians of the city, and an expression from them would probably have more weight than a recommendation from associations of this kind. If an effort is made to secure municipal inspection in the city of St. Joseph I would suggest that the city Board of Health be approached in the matter, and try and secure their co-operation.

Dr. Netherton: During this last spring I circulated a petition among the physicians, and each one of them signed it. It was addressed to the Mayor and the city Board of Health, asking that an inspector be appointed, a veterinary graduate. I think there were something like seventy-five signatures of physicians and that of quite as many laymen. It was referred to the Board of Health and was pigeon-holed. They did not seem to take any interest in it. They had a food inspector in this city up to last year, a layman who never did anything, and the office was abolished. This fact was alleged as a reason for their failure to act upon the petition which I presented. However, I think that in the coming spring, if we have the co-operation of the physicians and the Board of Health, we will have inspection established again, and it would be much easier if we could get this on a fee system. I do not know of a precedent for such a system, but lack of funds for such a purpose was urged by even those who were favorable to the propositon. But as I have already said, I think if the matter is agitated until the end of the fiscal year we can get some money appropriated and have the office re-established next spring.

Dr. Cooper: The only case I recall now regarding public sentiment relative to milk-inspection occurred in Iowa the past summer. Almost every town in that particular section has from one to three creameries, and some one had made complaint about a man having tuberculous cows who was selling milk to one of the creameries. It was found upon investigation that the man had two or three cows affected with tuberculosis. He told the inspector that another man a short distance from there who was milking from fifty to one hundred cows had a bad herd. This herd was examined, and thirty cows were condemned. The water was furnished by a spring surrounded by hills, and around this was his pasture, and in rainy weather the wash from the hills was mingled with the water of the spring. In the winter his stables are crowded very full, and are not very clean. Not long after the quarantine had been established a local clergyman wrote a letter to the State inspector asking him to lift the quarantine of the rich man's cows, but saying nothing of the other man. The owner of the cows was quite wealthy, and the clergyman's influence would outweigh that of any half-dozen physicians or veterinarians you could get together. Where you have such interference as that it of course takes some little time to control public sentiment or enlighten them as to the actual facts in the case.

Dr. Forbes: Before closing the discussion I would like to emphasize some of the remarks made by several of the participants in this discussion as to the necessity of getting before the public the need for a thorough and competent meat-inspection and milk-inspection. I think, also, that the suggestion of some members as to going a little slow should be considered. We should be content with a very small thing at first, and wait until the public has been educated to it before pushing the matter to any great extent. I think by so doing we will arrive at better results. The remark made by Dr. Cooper as to supplying tuberculous milk to creameries suggests two or three cases of which I read in a journal the other day, of which I made extracts, and as this feature of the subject had not been treated of in former papers to which I have listened at the meetings of this Association, I thought it would interest the members:

Rabinowitch and Kemper inaugurated experiments to discover if the milk of cows that had reacted to the tuberculin-test could be proven virulent, whether there were any clinical signs of tuberculosis or not. The milk of fifteen cows which had reacted was selected, and guinea-pigs were inoculated. Ten of the cows gave virulent milk, three of them showed clinical signs of the disease, and two others did not show any sign after three clinical examinations, made at intervals of three months. The authors conclude from this that a clinical examination alone is not sufficient to assure the innocuity of the milk, and that only the tubeculin-test should be relied upon.

Ostertag also made experiments in this direction at the instigation of the Minister of Agriculture, and his conclusions are somewhat opposed to those of the former experimenters. He submitted the milk to direct bacteriological examination, inoculated it into the peritoneum of guinea-pigs, after centrifugation by Obermuller's method, and finally administered it by injection. He first experimented with the milk of forty-nine cows, and continued for one month. The results were negative. Guinea-pigs killed from forty-six to sixty-eight days after were found free from tuberculosis. In a second series of experiments one guinea-pig inoculated into the peritoneum was killed seventy-one days after, and was found slightly tuberculous, but a second inoculated with the same sample presented no trace of infection; and, again, a third guinea-pig which had ingested 300 grammes of the same liquid remained healthy. Thus the milk showed virulence only in a slight degree, and was not potent enough to cause the disease by ingestion in a subject so sensitive to tuberculosis as the guinea-pig. Ostertag concludes from this that the milk from cows reacting to tuberculin, without presenting any clinical signs of tuberculosis, may be regarded as not dangerous.

Kuno Obermuller has made some experiments to show the possibility of tuberculous infection of butter. He recounts that his experiments, made in 1895, were unsuccessful from the fact that he inoculated animals that died from peritonitis after a few days. He attributed these accidents to the resorption of the fat, and to avoid this he had recourse to centrifugation, which separated the fat, the milky residue containing the bacilli. He submitted ten samples of butter bought in Berlin to experiment. Forty-one guinea-pigs received ½ to 2 c.c. of the liquid, which was equal to about 4 to 16 c.c. of butter. The animals were separated by threes, fours, and fives in disinfected cages, and were weighed and observed daily. Twenty-nine showed evidence of the disease, and twelve remained healthy. Peritonitis was not observed. The diseased animals became much emaciated and showed typical lesions at post-mortem examinations. Cultures were made from the bacillus found in the lesions, which confirmed the post-mortem diagnosis.

Seven out of ten samples of butter showed virulency, and the author concludes that the tubercle bacillus in butter is not as rare as has been announced.

Morgenroth demonstrated the presence of the bacillus in margarine, and he affirms that it is not by any means rare. In the preparation of the product a temperature of 105° F. is hardly exceeded. Ostertag remarks on this subject that the product is largely made with the fat of the mesentery and mediastinum, the precise places where are included the glands most affected with tuberculosis.

This opens up another phase in the danger to the human family of tubercular infection from the food-supply obtained from animals.

Dr. Wilson: On ante-mortem we frequently find cows where the udder is enlarged and contains matter and little abscesses, and I would like to ask the writer of this paper how he would diagnose whether this diseased condition arose from tuberculosis or was simply an inflammatory product.

Dr. Netherton: I think that where you have an inflammation due to mammitis the nodules are not as small as in tuberculosis, but I believe it would have to go to post-mortem before it could be determined positively. There is a cow out here now in one of these dairies—a nice Holstein cow—

and giving about six gallons of milk daily. On the right hind-quarter a little abscess developed. It commenced as mammitis, I think, and I prescribed the regular treatment for mammitis, to which it seemed to yield partially. The enlargement abated to about the size of a hen's egg. Two weeks later I was called again, and found it had become very hard. I put a knife in it and got quite a bit of pus. It seemed to heal up, and left a little nodule there about the size of a walnut. It ran along that way for four or five months, and did not seem to bother her, when about two weeks ago it commenced enlarging and it got the size of a goose-egg. I was again called and again applied the knife, and, as before, obtained a considerable quantity of pus. It is going down nicely, and I think will soon be all right, but all this time the milk from this cow has been sold in the city. I do not know whether it was a case of simple mammary abscess or tubercular abscess.

Dr. Peters: I would call it a suspicious case of tuberculosis. All such cases should be tested with tuberculin.

Dr. Stewart: As the question has drifted into one of ante-mortem and post-mortem determination, I might state that it is altogether probable that there are many cases of mammitis which result in what is popularly known as spoiled bag which are not tubercular. The udder, one or several quarters of it, may be involved, but is usually involved as a whole, and upon palpation presents quite a uniform surface, which is quite dense, and in these cases there is no characteristic enlargement of the lymphatic glands above and behind the udder, while in cases of tubercular mammitis tubercular masses are developed in the substance of the udder, usually so pronounced that they can be readily determined by palpation; also there is involvement of the supermammary lymphatic glands. I do not know that it is common to find a tubercular udder in which the tubercular process has resulted in the formation of abscesses such as have been described. However, there are so many things with which I am not familiar which are facts that I would not undertake to say the case described is not tubercular. Certain it is that it would be good policy to test a case of that kind with tuberculin, and in that manner pass upon the question.

Dr. Wilson: Down here we get many cases of that kind almost every day, in fact, and we pass them along unless they show other symptoms along with the enlargement, where the udder has had little abscesses form from spoiled bag, and upon post-mortem examination they show no sign or trace of tuberculosis in most cases.

Dr. Cooper: I had a case last summer at the packing-house at Kansas City, the first I have noticed. The udder showed quite a large abscess and tubercular indications all around it. I told the boys to be careful and save the entrails. When the animal was hung up it showed no great emaciation; lungs were found to be very badly tuberculous, and intestines, liver, and ovaries badly involved. In fact, the whole system was about as bad as I ever saw. She was not fat, nor yet what you would call thin, and probably might be passed as a cow with spoiled bag. This large abscess would probably hold half a teacupful in one side of the udder, on others not quite so much, of quite soft pus, which flowed readily, and which did not have the usual appearance of pus which you will see in tuberculosis—different color somewhat and a great deal thinner.

(To be continued.)

